# Prevalence and associated risk factors for Hepatitis B and C viruses among refugees in Gambella, Ethiopia

**DOI:** 10.1186/s12889-020-08893-1

**Published:** 2020-05-19

**Authors:** Abiyu Ayele, Dessie Abera, Melese Hailu, Muluken Birhanu, Kassu Desta

**Affiliations:** 1 Department of Medical Laboratory Sciences, Ethiopian Airport, Addis Ababa, Ethiopia; 2 Department of Medical Laboratory Sciences, College of Health Sciences, Addis Ababa University, Addis Ababa, Ethiopia; 3 Department of Medical Laboratory Sciences, College of Health Sciences, Assosa University, Assosa, Ethiopia

**Keywords:** HBV, HCV, Refugees, Gambella, Ethiopia

## Abstract

**Background:**

Currently, there is an increased flow of refugees into Ethiopia from neighboring countries. However, there are no post-arrival screening mechanisms for hepatitis B and C viruses which could be an additional burden for the local population. Hence, this study aimed to determine the prevalence and associated risk factors for hepatitis B and C viruses among refugees in Gambella, Ethiopia. It also aimed to determine the knowledge, attitude, and practice concerning hepatitis B and C viruses among participants.

**Methods:**

A cross-sectional study was conducted among 453 refugees in Gambella, Ethiopia from January until May 2018. A questionnaire was used to collect data on refugees’ socio-demographic, risk factors, and KAP of hepatitis B and C infections. Five milliliters of blood sample were collected from each participant and the serum was used for HBsAg and anti-HCV antibody screening rapid tests. Positive samples were further tested by ELISA method. Data were performed using SPSS version 20, and a *p*-value less than 0.05 was considered statistically significant.

**Results:**

The overall prevalence of HBsAg and anti-HCV among refugees was 7.3% (33/453) and 2.0% (9/453) respectively. Of these, 6.8% (25/370) and 1.4% (5/370) of females were positive for HBsAg and anti-HCV, whereas 9.6% (8/83) and 4.8% (4/83) of males were positive for HBsAg and anti-HCV. The age group of 18–29 and 30–41 years old were related to HCV infection (*P* = 0.003 and *P* = 0.020). However, proposed risk factors were not related to HBV and HCV infections. Knowledge assessment showed that 86.5% (392/453) did not know how HBV and HCV infections are transmitted, and 86.8% (393/453) had no information about the availability of HBV vaccine.

**Conclusion:**

This study showed intermediate prevalence of hepatitis B and hepatitis C virus in a large refugee camp in Ethiopia. The prevalence of hepatitis C virus was found to increase with age, but no other risk factor for either virus identified as significant. Refugees’ understanding of hepatitis B and C was very limited. This indicates the need for screening policy to be implemented and integrated with other health services and awareness creation about the infection in all refugee camps of Gambella.

## Background

Globally, hepatitis B virus (HBV) and hepatitis C virus (HCV) are major public health problems, particularly in developing countries [1]. The majority of the people, 40–80% living with chronic hepatitis B or C are unaware of their serostatus and they remain infectious to others [2]. Chronic liver disease, due to HBV and HCV continues to be the most challenging problem in economically poor countries [3].

Refugee populations are at risk of contracting HBV and HCV infections because of a lack of access to health care services and the inability to obtain information about the transmission and prevention ways of these infections from the media [4, 5]. In addition, social and cultural norms, sexual violence, ritual scarification, as well as separation from a spouse may increase the risk of getting hepatitis infections due to HBV and HCV. Furthermore, isolation and stress may also lead the refugees to engage in risky behavior, which escalates the risk of these infections [6]. Although the extent of transmission potential varies, HBV and HCV share a common mode of transmission, which are unsafe sexual intercourse, percutaneous or mucosal exposure to the blood or body fluids of an infected person, exposure to unscreened blood transfusions or unsafe injections in health care settings and by intravenous drug use [7]. The risk of HBV and HCV transmission is strongly associated with the origin from countries in which these infections are endemic, lack of information on hepatitis prevention, history of sharp injury, blood donation, family history of liver disease, tooth extraction, and tattooing [7].

About 350 to 400 million people are infected with HBV worldwide and it is the cause of death for 1 million people each year. Furthermore, 130 to 170 million people are infected with HCV worldwide, which causes about 350,000 deaths per year [8]. The World Health Organization (WHO) classifies countries, according to the hepatitis B surface antigen (HBsAg) prevalence, into low (< 2%), intermediate (2–8%), and high (> 8%) prevalence [9].

Ethiopia is classified under the geographical regions with intermediate viral hepatitis infections [10]. HBsAg prevalence in sub-Sahara Africa ranges from 8 to 15%, [11, 12], whereas the prevalence of HCV in sub-Saharan Africa ranges from 0.1 to 13.8% [13]. A meta-analysis report in Ethiopia indicated that the estimated prevalence of HBsAg and anti-HCV antibody was 7.4 and 3.1% respectively [14].

The flow of refugees into Ethiopia is increased from time to time and ranked the second most populated refugee asylum countries in Africa. The majority of them are originated from Somali, Eritrea, Sudan, and South Sudan, which have a high prevalence of hepatitis B and C [3, 15, 16]. For instance, a meta-analysis report from Sudan indicated that the prevalence of hepatitis B surface antigen (HBsAg) and HCV- antibodies were 12.07 and 2.74% respectively [17], and a study from South Sudan estimated the prevalence of HBV to be 11% [18]. Refugees cross the border of the country without any health screening and live in different refugee camps in close proximity to the local inhabitants. As a result, different diseases can be transmitted from refugees to the local population unless extended health education and preventive mechanisms are planned by the country. In addition, Ethiopian public health policies do not focus on screening refugees for hepatitis B and C viral infection (13). Therefore, early detection is crucial, to prevent transmission, progression of liver disease and to decrease morbidity and mortality. So, this study aimed to determine the prevalence and associated risk factors for hepatitis B and C viruses among refugees in Gambella, Ethiopia. It also aimed to determine the knowledge, attitude, and practice concerning hepatitis B and C viruses among participants.

## Methods

### Study design, period and setting

A cross-sectional study was conducted from January 2017 until May 2018 at Pugnido-I refugee camp in Gambella, Ethiopia. The study area is located in the southwest of Ethiopia and borders with South-Sudan. It is 711 km from Addis Ababa, the capital city of Ethiopia. In Gambella, there are six refugee camps, with a total number of 285,846 refugees, namely Pugnido-II (16,820), Nguenyyiel (28,243), Kule (51,272), Jewi (56,989), Pugnido-I (62,925) and Tierkidi (69,597). Inhabitants of Pugnido-I refugee camp were primarily originated from South Sudan. We have observed that adult male participants were very few in the camp because they have stayed in their country to fight.

### Study participants, sampling technique and sample size determination

A list of all the refugee camps in the study area was obtained from the administration for refugee and returnee Affair (ARRA), Addis Ababa, Ethiopia. Accordingly, there are six refugee camps, and from these refugee camps, Pugnido-I refugee camp was selected as a study site using a purposive sampling technique because of the availability of a well-organized laboratory and a huge number of refugees in the camp. After that, all refugees visiting Pugnido-I camp health center during the study period and who volunteered to participate in the study were enrolled conveniently.

The sample size was determined by using the single population proportion formula, *n* = (Zα/2)^2^ P (1- P)/ d^2^, with a marginal error of 5, and 95% confidence interval [19]. Data on the prevalence of HBV among refugees in the study setting was not available, however, it is expected to be in the range of 8–15% [20]. Therefore, we used 15% to increase the sample size, accordingly, the sample size was determined to be 196. To minimize errors arising from the likelihood of non-response, 10 % (10%) of the sample size was added giving a final minimum sample size of 216. Nevertheless, we have collected 453 samples.

### Data collection

Written consent was obtained from study participants and an interviewer-administered questionnaire was used to interview participants about their socio-demographic characteristics (age, sex, marital status, and educational status), and we also assessed their exposure to potential risk factors of HBV and HCV infections, such as the habit of sharing sharp materials, history of tattooing and other risks. In addition, knowledge, attitude, and practice towards HBV and HCV infections were assessed using yes/or no questions focusing on the cause, transmission, prevention and treatment of viral hepatitis due to HBV and HCV. The questionnaire was prepared in the English language and supervisors helped to translate for those who did not communicate in English. The cut- off value for adequate knowledge, attitude, and practice was defined as 70% or more questions were correctly answered. The questionnaire was developed and adapted after detail review of relevant literatures [21–24]. The English version of the questionnaire is supplied as a supplementary document (See Additional file 1).

### Laboratory analysis

After obtaining the participants’ written consent, 5 ml of the blood sample was collected from each refugee. Each blood samples were allowed to clot and serum was separated by centrifuging at 3000 rpm for 5 min and all serum samples were tested for HBsAg and anti-HCV rapid test strips (Eco test) according to the manufacturer’s instruction. All serum samples which were positive for HBV and HCV further tested with Enzyme-Linked Immunosorbent Assay (ELISA) at Ethiopian Public Health Institute (EPHI). During testing, standard operating procedures (SOPs) were followed for each laboratory analysis, and known positive and negative serums for HBV and HCV tests were used as a control to avoid false positive and negative results.

### Statistical analysis

Data were coded, entered, and analyzed by SPSS software version 20. Descriptive statistics were performed to describe the frequency of categorical variables, such as the socio-demographic characteristics, risk factors and knowledge, attitude, and practice, as well as to estimate the prevalence or proportion of HBV and HCV infections. The associations of potential risk factors with HBV and HCV infections were assessed using binary logistic regression. Moreover, all variables with a *P*-value < 0.25 in the bivariate analysis were included in the multivariate logistic regression model, to look if the association existed after controlling against all the rest of the variables, and a cut off value less than 0.25 is supported by the literature [25, 26]. Odds ratio (OR) at 95% confidence interval (CI) was calculated and a *p*-value < 0.05 was considered as statistically significant.

### Ethical approval

The study was approved by the Departmental Research and Ethics Review Committee (DRERC) of Addis Ababa University, Department of Medical Laboratory Sciences, Ethiopia. A letter of permission was obtained from the office of the Administration of Refugees and Returnees Affair (ARRA) and UNHCR, Ethiopia. Written informed consent was obtained from the study participants after they have been clearly briefed about the objective of the study. They were informed about their right to refuse to participate in the study and to withdraw at any time during the study without jeopardizing their right to access other health services. Test results were kept confidential by using unique codes given to each study participant. All positive results were communicated to the attending physician**.**

## Results

### Socio-demographic characteristics

During the study, 473 volunteer refugees gave their consent to participate. Nine participants were excluded because of the incompleteness of the questionnaire, and eleven hemolyzed blood specimens were rejected, with the total exclusion of 4.2% (20/473). Finally, 453 participants who gave both blood specimens and questionnaire responses were included in the analysis. The majority of the participants**,** 370 (81.7%) were females and the age range of the participants was 18–61 years old with a mean age of 29.6 ± 9.3SD years. Concerning marital status, 379 (83.7%) of them were married. Data on educational status showed, 166 (36.6%) were elementary level and roughly the same number, 165 (36.4%) were illiterate (Table 1).

**Table 1:** Socio-demographic characteristics of the study participants in Gambella, Ethiopia, 2018 (*n* = 453)

Variables		Frequency (n)	Percentage (%)
Sex	Female	370	81.7
	Male	83	18.3
Age category	18–29	231	51.0
	30–41	107	23.6
	> 41	115	25.4
Marital status	Single	25	5.5
	Married	379	83.7
	Divorced	22	4.9
	Widowed	27	6.0
Education	Illiterate	165	36.4
	Elementary	166	36.6
	High school	104	23.0
	Higher education	18	4.0

### The prevalence of HBsAg and anti-HCV antibody

Out of 453 participants, 7.3 and 2.2% were positive for HBsAg and anti-HCV rapid tests respectively. Nearly the same prevalence was found with ELISA, which was used as a confirmatory test, 7.3% (33/453) prevalence of HBsAg, and 2.0% (9/453) prevalence of anti-HCV. The detection rate of HBsAg and anti-HCV in female participants was 6.8% (25/370) and 1.4% (5/370), whereas it was 9.6% (8/83) and 4.8% (4/83) among males respectively. A slightly higher proportion of HBsAg (12.1%) was detected among increased aged groups (30–41 years old). The positivity rate for both HBsAg,7.7% (29/379), and anti-HCV, 2.4% (9/379) among married participants was higher than among participants with other marital statuses. Participants who were educated at elementary level showed a high prevalence of HBsAg 12.0% (20/166) and anti-HCV 3.0% (5/166) (Table 2).

**Table 2:** Prevalence of HBV and HCV with ELISA among study participants in Gambella, Ethiopia, 2018 (*n* = 453)

Variables		HBsAg		Anti-HCV		Total
		Positive (%)	Negative (%)	Positive (%)	Negative (%)	
Sex	Female	25 (6.8)	345 (93.2)	5 (1.4)	365 (98.6)	370
	Male	8 (9.6)	75 (90.4)	4 (4.8)	79 (95.2)	83
Age category	18–29	15 (6.5)	216 (93.5)	5 (2.2)	226 (97.8)	231
	30–41	13 (12.1)	94 (87.9)	3 (2.8)	104 (97.2)	107
	> 41	5 (4.3)	110 (95.7)	1 (0.01)	114 (99.99)	115
Marital status	Single	1 (4.0)	24 (96.0)	0 (0)	25 (100)	25
	Married	29 (7.7)	350 (92.3)	9 (2.4)	370 (97.6)	379
	Divorced	1 (4.5)	21 (95.5)	0 (0)	22 (100)	22
	Widowed	2 (7.4)	25 (92.6)	0 (0)	27 (100)	27
Educational status	Illiterate	7 (4.2)	158 (95.8)	4 (2.4)	161 (97.6)	165
	Elementary	20 (12.0)	146 (88.0)	5 (3.0)	161 (97.0)	166
	High school	5 (4.8)	99 (95.2)	0 (0)	104 (100)	104
	University	1 (5.6)	17 (94.4)	0 (0)	18 (100)	18

*HBsAg* Hepatitis B surface antigen, *ELISA* Enzyme Linked Immunosorbent Assay, *HCV* Hepatitis C virus

### Risk factors associated with prevalence of HBsAg

The proportion of HBsAg positivity was higher among male participants, 9.6% (8/83) than among female participants, 6.8% (25/370), however, the difference was not statistically significant (*P* = 0.364). Regarding the marital status, 7.7% (29/379) of the married participants were positive for HBsAg and 7.4% (2/27) of the divorced participants was positive for HBsAg. Data from educational status showed that 12.0% (20/166) positive for HBsAg were educated at the elementary level. Participants who had an exchange of sharp materials with others turned out to have a slightly higher positivity of HBsAg, 6.3% (12/106) compared to participants who had no exchange of sharp materials with others. About 6.1% (21/347) of the refugees who had multiple sex partners were positive for HBsAg. Exchange of sharp materials, marital status, educational status and other proposed risk factors were not associated with HBV infection (Table 3).

**Table 3:** Risk factors associated with prevalence of HBsAg using ELISA test among study participants in Gambella, Ethiopia, 2018 (*n* = 453)

Variables		Total (%)	HBsAg Positive (%)	COR	CI (95%)	***P***-Value
Sex	Male	83 (18.3)	8 (9.6%)	1.00		
	Female	370 (81.7)	25 (6.8%)	0.67	0.29–1.56	0.364
Age category	> 41	115 (25.4)	5 (4.3)	1.00		
	30–41	107 (23.6)	13 (12.1)	3.04	0.31–2.75	0.894
	18–29	231 (51.0)	15 (6.5)	1.54	0.18–1.56	0.258
Marital status	Single	25 (5.5)	1 (4.0)	1.00		
	Married	379 (83.7)	29 (7.7)	1.98	0.26–15.23	0.508
	Divorced	22 (4.9)	1 (4.5)	1.14	0.06–19.42	0.926
	Widowed	27 (6.0)	2 (7.4)	1.92	0.16–22.58	0.604
Education status	University	18 (4.0)	1 (5.6)	1.00		
	High school	104 (23.0)	5 (4.8)	0.85	0.09–7.81	0.892
	Elementary	166 (36.6)	20 (12.0)	2.32	0.29–18.45	0.424
	Illiterate	165 (36.4)	7 (4.2)	0.75	0.08–6.49	0.796
Multiple sex partners	No	106 (23.4)	12 (11.3)	1.00		
	Yes	347 (76.6)	21 (6.1)	0.50	0.23–1.06	0.072
Exchange of sharp materials	No	389 (85.9)	29 (7.5)	1.00		
	Yes	64 (14.1)	4 (6.3)	0.82	0.28–2.43	0.731
Tattooing history	No	435 (96.0)	32 (3.4)	1.00		
	Yes	18 (4.0)	1 (5.6)	0.74	0.09–5.74	0.774
Tooth extraction history	No	402 (88.7)	30 (7.5)	1.00		
	Yes	51 (11.3)	3 (5.9)	0.77	0.22–2.63	0.683
Vaccinated	No	444 (98.0)	32 (7.2)	1.00		
	Yes	9 (2.0)	1 (1.1)	1.60	0.19–2.27	0.658

*COR* Crude odds ratio, *CI* Confidence interval

### Risk factors associated with anti-HCV antibody prevalence

The proportion of anti-HCV (1.4%) among female participants was lower than male participants (4.8), but the difference was not significant (COR = 0.27, CI 95% 0.07–1.03, *P* = 0.055). Statistically, significant association could be seen in the age group of 18–29 and 30–41-years old participants with HCV infection (COR = 2.52, CI 95% =1.81–6.36, *P* = 0.002 and COR = 3.28, CI95% = 2.02–9.54, *P* = 0.017) compared to other age groups, respectively. Only 1.6% (1/64) anti-HCV positive participants had a history of exchange of sharp materials with others; however, no significant association with HCV infection was found (*P* = 0.794). In addition, multivariate logistic regression was performed to account for possible confounding variables and all variables with a *P*-value < 0.25 in the bivariate analysis were included in the multivariate logistic regression analysis. Accordingly, adjusted odds ratio was calculated between different age groups and sex and HCV infection. The association remains significant among the age category of 18–29 years (AOR = 2.47, CI 95% = 1.83–7.40, *P* = 0.003) and age category of 30–41 years old (AOR = 3.20, CI 95% = 2.78–8.11, *P* = 0.020), whereas sex was not significant with HCV infection (*P* = 0.083) as depicted in (Table 4).

**Table 4:** Risk factors associated with prevalence of anti-HCV antibody using ELISA test among study participants in Gambella, Ethiopia, 2018 (*n* = 453)

Variables		Total (%)	Anti-HCV Positive (%)	COR	CI (95%)	***P***-Value	AOR	CI (95%)	***P***-Value
Sex	Male	83 (18.3)	4 (4.8)	1.00			1.00		
	Female	370 (81.7)	5 (1.4)	0.27	0.07–1.03	0.055	0.29	0.07–1.17	0.083
Age category	> 41	115 (25.4)	1 (0.01)	1.00					
	30–41	107 23.6)	3 (2.8)	3.28	2.02–9.54	0.017	3.20	2.78–8.11	0.020
	18–29	231 (51.0)	5 (2.2)	2.52	1.81–6.36	0.002	2.47	1.83–7.40	0.003
Multi sex partners	No	106 (23.4)	2 (1.9)	1.00					
	Yes	347 (76.6)	7 (2.0)	1.07	0.21–5.23	0.933			
Exchange sharp Material	No	389 (85.9)	8 (2.1)	1.00					
	Yes	64 (14.1)	1 (1.6)	0.75	0.09–6.14	0.794			

*COR* Confidence interval, *AOR* Adjusted odds ratio

### Knowledge, attitude and practices (KAP) assessment on HBV and HCV infection

#### Knowledge of study participants

The majority of participants 79.2% (359/453) had no knowledge about HBV and HCV infections and 86.5% (392/453) and 91.4% (414/453) did not know about the transmission of HBV and HCV, respectively. About 89.0% (403/453) of the participants did not know the relationship between liver cancer and hepatitis B and C. Concerning vaccination, 86.8% (393/453) did not have any information about the availability of a vaccine against hepatitis B and 72.0% (326/453) also did not have information on the treatment of HBV and HCV infection at all (Table 5).

**Table 5:** Knowledge, Attitude and Practice assessment on HBV and HCV infection among study participants in Gambella, Ethiopia, 2018 (*n* = 453)

Knowledge assessment questions	Yes (%)	No (%)
Have you ever heard about HBV and HCV infection?	94 (20.8)	359 (79.2)
Is hepatitis transmitted through sex?	61 (13.5)	392 (86.5)
Can you get hepatitis infection through body fluid contact?	39 (8.6)	414 (91.4)
Do HBV and HCV cause liver cancer?	50 (11.0)	403 (89.0)
Is there a vaccine for HBV?	60 (13.2)	393 (86.8)
Is there effective treatment for HBV?	127 (28.0)	326 (72.0)
Is there effective treatment for HCV?	142 (31.3)	311 (68.7)
**Attitude assessment questions**		
Do you perceive that HBV & HCV can be transmitted through food?	37 (8.2)	416 (91.8)
Do you think hepatitis infection is a curable disease?	52 (11.5)	401 (88.5)
Do you think that hepatitis is a serious public health problem?	110 (24.3)	343 (75.7)
Do you think that taking HBV vaccine is safe?	123 (27.2)	330 (72.8)
**Practice assessment questions**		
Have you received HBV vaccination?	7 (1.5)	446 (98.5)
Have you ever been screened for HBV and HVC?	58 (12.8)	395 (87.2)
Have you exchange of intravenous drug use?	1 (0.2)	452 (99.8)

*HBV* Hepatitis B Virus, *HCV* Hepatitis C virus

#### Attitude of study participants

In this study, 8.2% (37/453) of the refugees believed that HBV and HCV infection could be transmitted by food and 11.5% (52/453) of the participants had the opinion that HBV and HCV are curable diseases. About 75.7% (343/453) of the participants thought that HBV and HCV are not serious public health problems and 72.8% (330/453) of the participants believed that the vaccine of HBV is not safe (Table 5).

#### Practice of study participants

Regarding the vaccination, 98.5% (446/453) were not vaccinated for hepatitis B. Furthermore, 87.2% (395/453) of the participants had never been screened for hepatitis B or C before (Table 5).

## Discussion

Hepatitis caused by hepatitis B and hepatitis C virus represents a widespread major health problem and it is very serious in the case of refugees that often live in circumstances that facilitate the spread of infectious diseases, such as HBV and HCV [5]. Globally, 45% of the population live in areas with high HBV prevalence, while another 40% live in regions with intermediate prevalence [27]. Although direct comparison is difficult because of limited published data in Africa, we have tried to compare our results with other high-risk groups and refugees found in other countries, but originated from Africa.

The prevalence of hepatitis B surface antigen (HBsAg) in the present study among refugees was 7.3%, which was classified as an intermediate prevalence [28]. The probable reasons for this intermediate prevalence might be due to a lack of knowledge about the transmission and the prevention of ways of the infection, having multiple sexual partners, and a large number of study participants were not vaccinated from their origin of country. Another possible explanation could be due to the origin of the country, the majority of them were from South Sudan, which has a high prevalence of HBV infection, 23.8% [29]**.** In addition, sexual promiscuity and scarification have been identified as common risk factors for HBV infection [30].

The current finding was much lower than reports from South Sudan among high- risk groups, 11% [18], and 26% [31]. Similarly, it was less than studies done in South Sudan, 12.3% [32], and central Sudan, 17.5% [33]. The variation could be due to differences in the study period, or sample size difference. Our result was congruent with a previous national pooled prevalence of 7.4%, in Ethiopia [14], and among pregnant women in Sudan, 7.5% [34]. In contrast, it was higher than previous studies in Ethiopia conducted at different risk groups: 3.0%, measured among women during delivery, 4.7% among blood donors, 6.0% among waste handlers, 4.2% in a military camp [35–38]. However, it was lower than studies done in Gondar among street dwellers, 10.9%, among young males in all regions of Ethiopia, 10.8% [39, 40], and South Sudan study among out patients in six clinics, 26.0% [30]. The above variations might be due to differences in study groups, level of exposure to the risks, test methodology, origin of country, and sample size.

The current study revealed a lower prevalence of hepatitis B than previous studies done in different African immigrants in the USA: Eritrean, 15.5%, Sudanese, 9.1% and Somalian, 8.3%, whereas it was higher than Kenya, 5.9%, Tanzania, 4.1% Burundi, 3.1% and Rwanda immigrants 3.0% [41]. Similarly, it was also lower than reports from immigrants in different parts of the world, such as from Bari-Italy, 8.3% [42], from South-Europe, 11.9% [43], among Afghan refugees in Pakistan, 8.3% [44], from India-Tibetan refugees, 8.9% [45]. This discrepancy might be related to the difference in test methods, larger sample sizes, study design and origin of the country. Our study showed higher prevalence of hepatitis B than previous studies among immigrants in different countries, UK, 5.0% [9], India, 6.2% [46], northern-Europe, 3.8% [47], and Kosovo, 2.9% [48]. The reasons for these variations might be due to variation in geographical distribution as well as population differences in terms of lifestyle and good awareness about the transmission and prevention of the infection.

The worldwide prevalence of hepatitis C is estimated to be 3.0% [3], with the highest prevalence 14.7% in Egypt [49]. According to the World health organization (WHO) classification, the prevalence of hepatitis C can be graded as high (> 3.5%), moderate (1.5–3.5%), and low (< 1.5%) [50]. Based on the above evidence, the current study revealed an intermediate prevalence of anti-HCV, 2.0%, which might be because of lack of knowledge about the transmission and prevention of HCV infection, lack of access to health care services, and the inability to obtain healthcare information from the media, or it could be due to the exposure of HCV in their country of origin.

This finding was congruent with an old study in Ethiopia among urban and rural populations, 2.0% [51], however, it was relatively lower than previous studies from South Sudan, 3% [30], and central Sudan, 2.2% [52]. This study was higher than studies conducted in Ethiopia at different risk groups: among medical waste handlers 1.0% (16) among military personnel 0.2% (18) and 0.2% among blood donors [53]; in contrast, it was lower than among patients on antiretroviral treatment (ART), 4.3% [54] and 5.8% prevalence among blood donors [55]. These variations could be due to differences in the study group, test methodology, study setting, and sample size.

The prevalence of hepatitis C in this study was lower than compared to refugees in different countries, such as Italy, 4.5% [42] and Greece, 2.3% [5], whereas it was higher than previous studies from Malta, 0.6% [46] and Germany, 1.9% [56]. The difference might be due to the difference in geographical location, origin of the refugees, and sample size.

There is no consensus on the role of gender as a risk factor for HBV and HCV infection [57]. The proportion of HBsAg positive rate was higher among male participants, 9.6% (8/83) than female participants, 6.8% (25/370). Similarly, anti-HCV positivity rate was higher among male participants, 4.8% (4/83) than female participants, 1.4% (5/370), however, the difference was not statistically significant (*P* = 0.083). This finding was comparable to a study conducted in Italy [42]. In contrast, a higher prevalence of HCV among females was reported in Yemen and Egypt [58, 59]. However, further studies on sex-specific prevalence of HBV and HCV are needed.

During this study, the prevalence of anti-HCV showed a significant relationship with being between 18 and 29 (*P* = 0.003) and 30–41 years old (*P* = 0.020), although the number of refugees positive for anti-HCV was quite limited, which limits the external validity of the *p*-value. This finding was consistent with studies conducted by Greenaway et al and Lu et al [56, 60]. This significant relationship might be due to being between 18 and 29 years old, and 30–41 years old implies being an adult, often sexually active, more risk of HCV. Marital status, educational status, exchange of sharp materials with other persons, multiple sexual partners were not associated with either HBV or HCV infection [45]. Lack of significant association might be due to small sample size, and only 9 persons who were positive for HCV, which means that the group of positivity is too small to find any significant and trustful relationship between sexual behavior, education and exchange of sharp materials, and the prevalence of HCV and HBV.

The current study assessed participant’s knowledge, attitude, and practices about HBV and HCV infection. A huge lack of knowledge was observed among refugees about viral hepatitis, its mode of transmission, and the possibility of prevention by vaccination. This was consistent with a study by Alemairy et al in Sudan among barbers and women hairdressers, which reported below 50% [61]. However, it was inconsistent with previous studies in Sudan among health care workers, which reported, 58.2% had an average level of knowledge [62], and greater than 80% knew the transmission and the prevention mechanisms of HBV infection [63]. The variation might be due to educational level difference, the majority of our study participants were illiterate and elementary level, while in their studies the participants were health care workers, which have a relative advantage of health care services and information about HBV infection transmission and prevention ways. It was also incomparable to studies from Asian American immigrants and Chinese immigrants showed, 61 to 90% of participants knew that HBV and HCV infection can transmit through body fluid contact and unsafe sex, moreover the participants had information about HBV vaccination [64, 65].

With regard to refugees’ attitudes, 8.2% (37/453) have perceived that HBV and HCV infection can be transmitted by food. Similarly, studies conducted among Korean and Cambodian immigrants in the USA, revealed that food can be a source of hepatitis B transmission [66, 67]. Almost seventy- 3 % (72.8%) of the participants believed that a hepatitis B vaccine is not safe. In contrast, a study among Chinese immigrants in the USA have a positive attitude towards vaccination [66, 68].

In terms of refugees’ practice, our result showed that there was a huge gap concerning screening and vaccination among the majority of the investigated refugees. Moreover, there was no routine or occasional vaccination of adults against HBV in the camp. This finding was incomparable to the previous study among immigrants in British stating that 71% screened for HBsAg and 44% received both testing and vaccination [69].

### Limitations of the study

Financial reasons made it impossible to perform all diagnostic markers of hepatitis, which would have been helpful to differentiate chronic infection from acute infections and to determine viral load. In addition, small sample size in our study might limit the association of risk factors with HBV and HCV, and the cross-sectional nature of this study makes it difficult to attribute causality to the observed associations. Furthermore, the representativeness of this finding is limited because our study conducted only in one refugee camp. Therefore, large scale longitudinal study design is needed for the future.

## Conclusion

In general, the prevalence of hepatitis B and C viruses was intermediate among study participants at Pugnido-I Camp refugee in Gambella, Ethiopia. The prevalence of hepatitis C virus was found to increase with age, but no other risk factors for either virus were identified as significant. Refugees’ understanding of hepatitis B and C was limited. This indicates the need for screening policy to be implemented and integrated with other health services and awareness creation about the infection in all refugee camps of Gambella, Ethiopia.

## Supplementary information

**Additional file 1:** A questionnaire used for collect data on socio-demographic, risk factors, knowledge, attitude, and practice of refugees towards HBV and HCV infections.

## Data Availability

The datasets used and/or analyzed during the current study are available from the corresponding author on reasonable request.

## Abbreviations

HBV: Hepatitis B virus
HCV: Hepatitis C virus
HBsAg: Hepatitis B surface antigen
WHO: World Health Organization
KAP: Knowledge, Attitude and Practice
