# Supplementary material for: Prevalence and associated risk factors for Hepatitis B and C viruses among refugees in Gambella, Ethiopia
Source: BMC Public Health. 2020 May 19;20:721. doi: 10.1186/s12889-020-08893-1 (PMC7236441; doi:10.1186/s12889-020-08893-1)
Supplement: Supplementary file 1 — Additional file 1: A questionnaire used for collect data on socio-demographic, risk factors, knowledge, attitude, and practice of refugees towards HBV and HCV infections. [file 12889_2020_8893_MOESM1_ESM.docx]

## Questionnaire for HBV and HCV related risk factors and KAP assessment

This questionnaire is designed to assess the socio-demographic, associated risk factors and KAP of HBV and HCV infections in refugees at Pugnido-I refugee camps Gambella, Ethiopia.

Participant Code no-------------------

1. **Socio-demographic of the participant (circle your answers)**

| **No** | **Question** | **Response Option** |
| --- | --- | --- |
| 1. | Sex | 1.Female 2.Male |
| 2. | Age category (years) | 1. 18-29 2. 30-41 3. >41 |
| 3. | Marital status | 1. Single 2. Married 3. Divorced 4.Widowed |
| 4. | Education | 1. Illiterate 2. Elementary 3. High school,  4. Higher education. |
| **B. Associated risk factors of hepatitis B and C viruses infection** | | |

| 1 | Do you have history of multi sexual partner in life? | 1. Yes. 2.No. |
| --- | --- | --- |
| 2 | Do you have sharing of sharp materials with others? | 1. Yes. 2.No. |
| 3 | Do you have history of tattooing? | 1. Yes. 2.No. |
| 4 | Is there family history of liver disease? | 1. Yes. 2.No. |
| 5 | Do you have history of tooth extraction? | 1. Yes. 2.No. |
| 6 | Have you ever taken HBV vaccination? | 1.Yes 2.No |

**C. Participant knowledge towards HBV and HCV infection**

| **No** | | **Question** | **Response Option** | | **Remark** |
| --- | --- | --- | --- | --- | --- |
| **1** | | Have you ever heard about HBV and HCV infection?iiiiIinfectionhepatitis/livdisease/? | **1.yes 2.No** | |  |
| 2 | | Is Hepatitis transmitted through sex? | 1. Yes. 2. No. | |  |
| 3 | | Can you get hepatitis infection through body fluid contact? | 1. Yes. 2. No. | |  |
| 4 | | Do HBV and HCV cause liver cancer? | 1. Yes. 2. No. | |  |
| 5 | | Does HBV have vaccine? | 1. Yes. 2. No. | |  |
| 6 | | Is there effective treatment for HBV and HCV? | 1.Yes 2.No | |  |
| **D. Participants attitude towards HBV and HCV infection** | | | | | |
| 1 | Do you believe HBV &HCV transmitted through food? | | | 1. Yes 2. No |  |
| 2 | Do you think hepatitis infection is curable disease? | | | 1. Yes 2. No |  |
| 3 | Do you believe hepatitis infection is serious public health problem  problemproblem? | | | 1. Yes 2. No |  |
| 4 | Do you think taking HBV vaccine is safe? | | | 1. Yes 2.No |  |
| **E. Participants practice towards HBV and HCV infection** | | | | | |
| 1 | Have you received HBV vaccination? | | | 1. Yes 2. No |  |
| 2 | Have you screened for HBV and HVC? | | | 1. Yes 2. No |  |
| 3 | Have you exchange of injection with drug users? | | | 1. Yes 2. No |  |
